# Integrated Care Programs for People with Multimorbidity in European Countries: eHealth Adoption in Health Systems

**DOI:** 10.1155/2020/9025326

**Published:** 2020-04-08

**Authors:** Maria Gabriella Melchiorre, Roberta Papa, Sabrina Quattrini, Giovanni Lamura, Francesco Barbabella, on behalf of ICARE4EU Consortium

**Affiliations:** Centre for Socio-Economic Research on Ageing, National Institute of Health and Science on Ageing, IRCCS INRCA, Via S. Margherita 5, 60124 Ancona, Italy

## Abstract

**Introduction:**

eHealth applications have the potential to provide new integrated care services to patients with multimorbidity (MM), also supporting multidisciplinary care. The aim of this paper is to explore how widely eHealth tools have been currently adopted in integrated care programs for (older) people with MM in European countries, including benefits and barriers concerning their adoption, according to some basic health system characteristics.

**Materials and Methods:**

In 2014, in the framework of the ICARE4EU project, expert organizations in 24 European countries identified 101 integrated care programs. Managers of the selected programs completed an online questionnaire on several dimensions, including the use of eHealth. We analyzed data from this questionnaire, in addition to qualitative information from six innovative programs which were studied in depth through case study methodology, according to characteristics of national health systems: a national health model (financing system), overall strength of primary care (PC) (structure/service delivery process), and level of (de)centralization of health system (executive powers in a country).

**Results:**

85 programs (out of 101) adopted at least one eHealth tool, and 42 of these targeted explicitly older people. In most cases, Electronic Health Records (EHRs) were used and some benefits emerged like improved care management and integration, although inadequate funding mechanisms represented a major barrier. The analysis by health system characteristics showed a greater adoption of eHealth applications in decentralized countries, in countries with a National Health Service (NHS) model, and in countries with a strong/medium level of PC development.

**Conclusions:**

Although in the light of some limitations, findings indicate a relation between implementation of care programs using eHealth tools and basic characteristics of health systems, with decentralization of a health system, NHS model, and strong/medium PC having a key role. However adaptations of European health systems seem necessary, in order to provide a more innovative and integrated care.

## 1. Introduction

Chronic diseases—like cardiovascular diseases, diabetes, cancer, and chronic respiratory diseases—represent the main cause of functional impairment and mortality in many countries [1], with 85% of related deaths in Europe [2]. Moreover, in the European region, about 70-80% of healthcare budgets are spent on chronic diseases [3], of which 97% on treatment and only 3% in prevention [4]. A great number of people (about 50 million) are also suffering from multimorbidity (MM) that is defined as any cooccurrence of multiple chronic conditions (MCCs) within one person [5]. MM prevalence is high especially among older people, with around 60% of patients aged 65+ [1, 6] and 82% aged 85+ years [7] living with MCCs. Furthermore, MM estimates vary across countries. Nielsen and colleagues [8] found the lowest MM prevalence of about 26% in Northern Europe and, the highest one, about 35%, in Central and Eastern Europe. A previous study also found a higher prevalence of MCCs in Eastern European countries compared with those in the Western area [9]. MM estimates in older adults also vary according to the different data sources [10].

National health systems in Europe still approach the complex health and social care needs of patients with MM focusing on traditional single-disease-oriented care programs, running mostly at a local level and without wider coordination [1, 11]. A new organization of care seems needed for addressing appropriately the challenge of MM, which requires proactive integrated initiatives, especially for older people [12]. In this respect, eHealth solutions—i.e., the application of innovative Information and Communication Technologies (ICTs) in the healthcare sector [13]—have the potential to provide new tailored integrated care services to patients with MM, also supporting patient centeredness, self-management, and multidisciplinary care [14, 15]. In fact, eHealth tools can offer to patients with MM relevant improvements for accessing personalized healthcare services and can enable new opportunities for treatment, rehabilitation, and maintaining healthy lifestyles and well-being [16]. The “promise” of eHealth is thus almost comprehensive, with better quality services and more efficient and effective care [17]. eHealth and information systems also can improve quality of available health data, in order to better assess efficiency of care itself [18].

In particular, the adoption of ICTs in health services and processes can innovate the provision of care at distance [19, 20], especially useful for older people living in the community [21, 22]. ICT has also been identified as a crucial enabler for supporting information sharing across health professionals [23, 24]. Some authors [25, 26] highlighted indeed that MM is associated with a more intense use of eHealth for information and communication purposes regarding health-related services.

The European Commission has supported member states in developing their eHealth strategies for more than a decade. This process started with the first *Action Plan eEurope 2002* that contributed to promote awareness and implementation strategies of eHealth across Europe [27] and continued with the *eHealth Action Plan 2012-2020* [28]. The latter put issues of chronic care and MM as policy priorities at a European level, aimed at utilizing eHealth for improving chronic diseases and MM management and prevention, with a crucial role in structural reforms which are necessary to ensure the sustainability of health systems. Previous European policies focused mainly on stimulating the general implementation of electronic health records (EHRs) and health information networks, in order to improve health data exchange between different care providers and nations [29]. The recent Communication from the European Commission [30], on enabling the digital transformation of health and care in the European Union's Digital Single Market, in particular highlights personalized medicine, citizen empowerment, and secure/safe access to electronic data as priorities of eHealth.

Nevertheless, eHealth tool implementation in Europe is not yet widespread. In most European countries, they have been somehow adopted by health systems but are not yet included in integrated care programs for patients with MM. In particular, we have a greater use of patient's EHRs [31]. A survey carried out in 31 European countries [32] showed that 93% of general practitioners (GPs) reported having an EHR system, although doubts on privacy and confidentiality of electronic health information prevent some GPs from utilizing such a tool. Literature also highlights that EHRs have a peculiar relevance to MM given that they allow healthcare providers to access electronic clinical information of patients with MM, which are characterized by multiple care providers, various health conditions and medical diagnoses, and potential drugs interactions [1, 25]. Currently, many countries have provided patient access to their EHRs, but legislations and policies regulating this right are greatly variable within nations [33]. Besides EHRs, across Europe, we find also the use of remote monitoring and consultation by means of telehealth services [34, 35], independent living solutions (e.g., assistive and ambient intelligence technologies) [36], and support for the family carers, especially of older people [37, 38]. A more recent survey on the global status of eHealth in the WHO European Region [39] reported that 70% of covered countries have a national eHealth policy, 59% have a national EHR system, and 80% have a national legislation protecting the privacy of EHRs.

The use of eHealth tools seems to show several potential benefits to patients with MM [31, 38], at the organizational level (e.g., better coordination/integration between professionals) and at the individual level (e.g., better monitoring of care, patient empowerment, and adherence to treatments). Some evidences [4] in particular indicated that inclusion of eHealth tools in integrated approaches has the potential to increase safety and quality of care for patients, by providing continuity across health and social services. However, there are various potential barriers hampering the implementation of eHealth technologies targeting people with MM [36, 40], such as lack of legislative regimes, lack of dedicated/adequate funding, limited privacy/ethics policies, and low adequate ICT infrastructures; also, cultural resistance to adopt technology both by patients and professionals [14], and lack of interoperability between eHealth applications [41] represent further barriers.

Although access to and use of digital technologies by patients are improving across Europe, national contexts are still rather different in terms of availability of ICT infrastructures, services, and skills among populations [29, 42]. Regarding geographical differences in adopting eHealth technologies, on the whole, we find the greatest use in Nordic countries (e.g., Denmark, Norway, Finland, and Sweden), whereas Southern and Eastern Europe include the lesser performing countries, with some exceptions, e.g., Spain [43, 44]. In particular, Denmark is the most advanced country in terms of eHealth adoption, with almost all doctors using electronic transfer of data and online exchange of patients' health data (91% of doctors exchanging EHRs, against 34% on average in other countries). Regarding on line appointments with health care practitioners, in 2016, this regarded 13% of EU residents, with 49% in Denmark, 35% in Finland, and 30% in Spain [45]. The Digital Economy and Society Index (DESI) [46] also indicates Finland and Denmark as high performing European countries with regard to eHealth in public service dimension.

The implementation of eHealth in European countries seems particularly depending on characteristics of health systems, which are defined by WHO ([39], p. 96) as “the ensemble of all public and private organizations, institutions and resources mandated to improve, maintain or restore health”. In this respect, Codagnone and Lupiañez-Villanueva [32] suggested that the context of the national health models represents a crucial aspect. Specifically, these authors identified three models of health system financing in Europe: the National Health Service (NHS), funded primarily by taxation; the Social Insurance System (SIS), funded through social insurance schemes; and the Transition Country (TC) system, usual in those Centre-Eastern European countries with postsocialist welfare system that joined the European Union in 2004-2007, which have still health systems in transition. A significant positive relation between high adoption rates of eHealth and the NHS model was found previously [32]. This result was further confirmed by Brennan and colleagues [47] with regard to the adoption of ePrescriptions (i.e., electronic drug prescription) in primary care (PC). They also found that the highest adoption rates occur in countries mainly belonging to the Nordic area and with the NHS model, whereas most of SIS and TC countries were in the middle/lower ranks. These results are also consistent with those regarding the wider implementation of eHealth in Europe, i.e., the NHS countries scored higher on the overall index [32, 47].

Moreover, a strong PC development (e.g., with strong service delivery process and structure) [48] seems linked to the growing possibilities of technology [49]. PC is indeed the first “entry-point” to the health system in many European countries, with a crucial role in coordinating patients throughout the different health settings [50], and a condition for its efficient work is the use of ICT. Living in countries with a strong PC system is in turn beneficial to people with MM [51], as the principles of PC—e.g., continuous, comprehensive, and coordinated care—may fit their needs in a better way [52, 53].

Furthermore, the level of (de)centralization of health systems, at a national or regional/local level of decision making and executive powers in a country, might impact the adoption of eHealth initiatives in different countries [54, 55]. In this respect, Saltman and colleagues [56] reported some positive outcomes regarding decentralized health systems, including increased capacity to innovate service delivery, with increased autonomy of local governments/institutions. Moreover, in countries where the responsibility for the provision of healthcare is decentralized, positive strategy documents regarding eHealth have been published by regional authorities [57]. Furthermore, different types of MM care practices may also be found in centralized and decentralized health systems [52]. Some authors also report that inequalities in ICT use are related not only to inequalities in individual social structures (e.g., sociodemographic, economic, and health variables) but also to macrosocial variables and welfare systems, e.g., economic, political, and social characteristics and public policies of the respective countries [58, 59].

The organization of a country's health care system can thus significantly impact eHealth diffusion [60]. Health systems represent the outcomes of health policy decisions, which in turn indicate the interrelationship between healthcare systems and health policy actors [61]. Regarding the selection of appropriate indicators to analyze health systems, Gauld [62] and Reibling [63] suggested, among others, the dimensions of “information technology” and “medical technology”, respectively.

On the basis of these considerations, the aim of this paper is to explore how widely eHealth tools have been currently adopted in integrated care programs for people with MM in Europe, according to three characteristics of health systems: the type of national health model (NHS, SIS, and TC), strength of PC development (strong, medium, and weak), and level of (de)centralization of a health system. We use this approach in order to explore whether eHealth is more adopted in countries with particular health system characteristics. Torrent-Sellens and colleagues ([64], p. 14) also put in evidence the need for “more in-depth research to be conducted into the link between eHealth usage and predictors, and the different health care systems in Europe”. We expect to find a greater implementation of programs with eHealth solutions in decentralized countries than centralized, with a strong PC than low, and with a NHS model than SIS and TC. Our research questions are thus the following:

- What types of integrated care programs for MM adopting eHealth have been adopted in (groups of) European countries, according to basic characteristics of health systems (national health model, strength of PC, and centralized/decentralized health system)?
- What categories of eHealth tools have been adopted in the integrated care programs, according to basic characteristics of health systems?
- What benefits/barriers of eHealth tools emerged in the integrated care programs, according to basic characteristics of health systems?

The responses to these questions might also suggest options/implications which could be of help for policy makers in facilitating the use/development of eHealth technologies within integrated care across different European health systems.

## 2. Materials and Methods

The care programs which are analyzed in this paper come from the Project “Innovating Care for People with Multiple Chronic Conditions in Europe” (ICARE4EU). This project (2013-2016) mapped innovative and integrated care approaches for people with MCCs, which have been implemented in 31 European countries, with the aim to increase and disseminate knowledge of European integrated care programs addressing MM. Below, information on materials and methods is reported. A more detailed description of these aspects is provided elsewhere [52, 65, 66].

### 2.1. Inclusion Criteria of the Programs

Programs were considered for inclusion in the survey when meeting the following criteria:

- Targeting adult people (aged 18 and older) with MM, defined as two or more medically diagnosed chronic or long-lasting (at least six months) diseases, of which at least one has a (primarily) somatic/physical nature
- Including formalized collaborations between at least two services
- Involving one or more medical services
- Being evaluable/evaluated
- Being, at the time of the survey (i.e., 2014), running, or finished in the previous 24 months, or starting within the following 12 months

### 2.2. Data Collection

Information on programs was collected with the support of expert organizations/program managers in each of the 31 countries of the European region included in the study. A list of potential country experts—with expertise on MM care and who can provide reliable information on innovative, multidisciplinary care approaches/programs for people with MCCs—was constructed for each country. They were asked to identify existing integrated care initiatives at a national/regional/local level focusing on MM and to report the related information by filling in an online questionnaire for each eligible program, also with the collaboration of their expert network and program managers/leaders. The online questionnaire was available in eleven languages and contained general questions (e.g., information on the target group of patients, main objectives and diseases addressed by the program, and quality and evaluation of the program) and specific aspects of MM care: patient centeredness, management practices and professional competencies, financing mechanisms, and use of eHealth technologies eventually adopted within the programs. According to these inclusion criteria, the country experts identified 101 programs on the whole, of which 85 are using at least one eHealth tool, from 24 European countries (out of 31 countries surveyed).

Moreover, eight good practices (or High Potential Programs (HPPs)) were selected for a more in-depth analysis, including site visits for qualitative data collection. For this purpose, the project team scored the 101 programs against five dimensions (general score, e.g., aim of the program, its strengths and weaknesses; level of patient centeredness; level of integration of care; innovativeness in financing mechanisms; and use of eHealth technologies) and thus, they identified the “top” eight HPPs to be further explored as case studies. These programs were operational in Belgium, Bulgaria, Cyprus, Denmark, Germany, Finland, the Netherlands, and Spain. Site visits were organized to study more in depth organizations, integrated care programs, ordinary activities, and relationships. Information was gathered by interviewing program managers and key care professionals from various disciplines/services, by using a topic guide-questionnaire in addition to relevant program documents if available (e.g., interim or final reports, program evaluations). The results of these visits were edited following a common template and are described in eight case reports that were published on the ICARE4EU website (http://www.icare4eu.org). For this paper, we only analysed information from the six (out of eight) HPPs that include aspects of eHealth.

For this paper, we also gathered information on health system characteristics of (groups of) countries which were included in the survey, such as national health models, (overall) strength of PC, and level of (de)centralization of health system. These aspects are detailed better below (Measures).

### 2.3. Ethical Aspects of the Study

In order to carry out the ICARE4EU study, no ethical approval was requested, given that there were no issues concerning privacy and anonymity of respondents. We provided indeed a protected web survey (by setting individual access credentials) to collect secondary data already available to country experts/managers and staff of integrated care programs for people with MM, without approaching patients or/and their caregivers. We thus collected various data only on the programs and not regarding personal/clinical/sensitive issues on patients and family carers. We signed anyway a written agreement with these experts/managers regarding the aim of the project and the dissemination of the anonymous/confidential data collected. Regarding the site visits to selected initiatives, all interviews were conducted by members of the ICARE4EU project team and were administered to experts/managers/leaders of the care programs. Also in this respect, only general (nonconfidential and nonpersonal) data on the programs was collected. Patients and their family caregivers were not approached by the project team. We signed a further written agreement with all these interviewees regarding the aim of the site visit, the consent to tape recording the interviews, and to publish the related case reports once validated and approved (including the name of the eight selected programs).

### 2.4. Measures

Our study firstly identified some general characteristics of care programs such as main objectives (e.g., increasing multidisciplinary collaboration, improving patient involvement), organizations involved (e.g., PC, general hospital), care providers involved (e.g., GP, medical specialist), integration level (e.g., small scale program, well-established program), operational level (e.g., policy/management, daily patient care), adoption level (e.g., local, regional, and national), geographical coverage (e.g., rural, urban), and types of care and support provided by programs (e.g., medical care, nursing care).

Our study then identified four categories of eHealth applications according to their main functions and adopted a classification by adapting key elements of the conceptual framework from the Chronic Care Model (CCM) and the eHealth Enhanced Chronic Care Model (eCCM) [29, 65, 67]. The four types of eHealth we identified are ICT tools for

- *Remote Consultation*, *Monitoring*, *and Care*: regarding remote/at distance interaction between patients and health professionals, e.g., consultations/visits by telehealth/telecare/telemedicine, online clinical appointments, and ePrescriptions
- *Self-Management*: regarding health advice and reminders used by patients to live more independently and with improved ability to self-care, e.g., computers, tablets, mHealth, and wearable devices/assistive technologies
- *Healthcare Management*: for improving the integration/communication, quality/efficiency of care processes within/between care providers, e.g., EHRs and health information systems on individuals shared between professionals; Personal Health Records (PHRs) managed by patients; and eReferral systems
- *Health Data Analytics*: systems for analysing clinical data/evidences regarding patients for prevention, monitoring, and treatment purposes, e.g., online decision supports used by health professionals for clinical decision making

Moreover, opinions (agreement *vs.* disagreement) on potential benefits (improving quality of care, quality of life of patients enrolled, integration/management of care, and cost efficiency) and barriers (inadequate legislative framework, funding, ICT infrastructures, and technical-ICT support; lack of skills and cultural resistance among care providers and patients; uncertainty about cost efficiency; compatibility/interoperability between different eHealth tools; and privacy/security issues) were addressed, as they were perceived by expert organizations/program managers.

In order to explore all the abovementioned aspects regarding programs adopting eHealth, according to basic information on health system characteristics of (groups of) 24 countries where integrated care programs were identified, the following dimensions were included (see Table S1 in the Supplementary Materials for more details on health system characteristics and countries/groups of countries):

- *National health model*: this classification is based on Codagnone and Lupiañez-Villanueva [32], concerning a WHO study measuring progress in eHealth adoption by GPs between 2007 and 2013. It distinguished countries with regard to their financing system of health care, as follows (and as already anticipated in Introduction of this paper): NHS (funded primarily by taxation), SIS (funded through social insurance schemes), and TC (including former Eastern Bloc countries with health systems in transition). This classification was further used by Brennan and colleagues [47], with specific regard to eHealth adoption
- (*Overall*) *strength of PC*: this classification is based on Kringos and colleagues [49, 68] and Detollenaere and colleagues [50] who analyzed data from 2009 to 2010. These data were collected as part of the European Union-funded project “Primary Health Care Activity Monitor for Europe - PHAMEU study”. In particular, Detollenaere et al. [50] based his own study on the framework (selection of the indicators, data collection, and calculation of the scales) described by Kringos and colleagues [49, 68], who distinguished countries with regard to strength (strong, medium, and weak) of their PC. Data included information/subdimensions on both PC structure (governance, e.g., policy implementation; economic conditions, e.g., expenditure/incentives systems; and workforce development, e.g., profile of professionals providing PC) and PC service delivery processes (accessibility, e.g., geographical distribution of services; comprehensiveness, e.g., available medical equipment; continuity, e.g., patient-GP relationship; coordination, e.g., gatekeeping role for GPs; and teamwork). Different combinations/developments of these indicators/dimensions correspond to/measure different degrees in the strength of PC between countries. For this paper, we considered the overall strength of PC
- *Level of (de)centralization of health system*: this classification is based on WHO, Health System in Transition series (years from 2008 to 2013) [69], and it distinguished countries with centralized health systems (most of the responsibilities lie with the central government) *vs.* decentralized systems (management systems whose regulation, operation, and also cofunding are delegated to regional authorities or states), as level of decision making and executive powers in a country [52]. This classification was derived from descriptive data in countries' latest Health System Review (in 2013, i.e., year when the ICARE4EU project was initiated).

### 2.5. Data Analysis

We have firstly analyzed the 101 integrated care programs targeting people with MM, using descriptive/quantitative frequency distribution, with regard to both their adoption of eHealth tools (at least one) and related distribution in 24 European countries. Then, we analyzed the bivariate relations between some aspects of programs adopting at least one eHealth tool—general characteristics, type of eHealth tools used, and (reported) potential benefits and barriers—and health system characteristics of respective groups of countries. For this purpose, we grouped the 24 European countries (where integrated care programs were identified) according to the country dimensions mentioned above, i.e., national health model, strength of PC, and level of (de)centralization of health system. We further grouped SIS and TC countries, in order to analyze programs in countries (predominantly) tax based *vs.* insurance based/mixed [52]. Moreover, we further grouped countries with strong/medium PC, in order to analyze them *vs.* countries with a weak PC system. The aim itself of the related PHAMEU study (cited above as basic framework for PC systems) was indeed to explore if “countries with relatively strong primary care have better overall health care system outcomes compared to countries with relatively weak primary care” ([49], p. 114). We thus decided to have only two groups of countries regarding the strength of PC, by integrating data on overall medium PC with data on overall strong PC, i.e., with the group of countries with the higher number of integrated care programs adopting eHealth in our study (Table 1).

It should be specified that in our analysis, the three health system characteristics are considered separately, without including a potential multidimensional relation between them. Anyway, additional analyses *(data not shown in Tables)* showed that out of 85 care programs with eHealth, 46 were implemented in decentralized countries with strong/medium PC too, and, among these, 37 were identified where NHS models were operating, whereas 22 programs were implemented in centralized countries with weak PC too, and, among these, 19 were however identified where NHS models were operating. Quantitative analyses were carried out with the statistical software SPSS 23.0. Bivariate analyses were performed by means of a *χ*^2^ test (chi-squared). The significance level for all analyses was set at *p* ≤ 0.05 (bold values within the Tables).

As a second step, we gathered/analyzed qualitative information from six site visits to HPPs adopting eHealth. For this purpose, we classified also the six good practices with regard to the abovementioned health system characteristics of the respective countries. Then, we provided additional insights with regard to benefits and barriers for using eHealth tools which were reported by program managers/other health professionals and referred to their ordinary/routinary care delivery to people with MM. The qualitative data analysis was performed by using a manual coding process [70], leading to conventional content analysis [71].

## 3. Results

### 3.1. Integrated Care Practices/Programs Using eHealth Applications in European Countries

**Figure 1:**
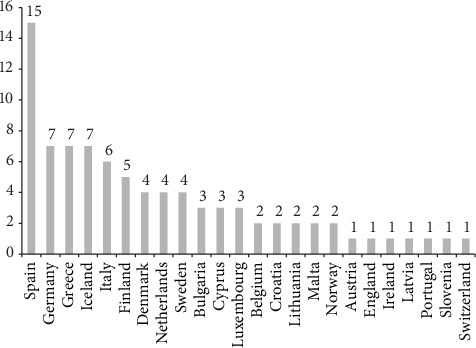
Number of integrated care programs using at least one eHealth tool by country (*N* = 85)^a^. ^a^The programs (on the whole and using at least one eHealth tool) were identified in the following 24 European countries: Spain, Greece, Iceland, Germany, Italy, Finland, The Netherlands, Denmark, Sweden, Luxembourg, Bulgaria, Cyprus, Belgium, Croatia, Malta, Lithuania, Norway, Ireland, England, Austria, Portugal, Slovenia, Latvia, and Switzerland. No eligible program was identified in Romania, Czech Republic, Hungary, Poland, Slovakia, and Estonia. Information on French programs was incomplete and thus excluded from the analysis.

Among 101 programs identified in 24 countries by the ICARE4EU project, 85 included the use of at least one eHealth tool (Figure 1), and of these, 42 targeted specifically older people aged 65+. The highest numbers of programs with eHealth adoption were identified in Spain (15); Greece, Iceland, and Germany (7 in each country); Italy (6); and Finland (5). In seven countries (e.g., Portugal, Slovenia, and Latvia), only one program using at least one eHealth tool was found. A more detailed description of general findings is reported in other publications [29, 65].

### 3.2. Number of Programs Using at Least One eHeath Tool and Health System Characteristics

As reported in Table 1, adoption of at least one eHealth tool in the programs, by health system characteristics, such as the national health model, strength of PC, and (de)centralization level of health system, showed on the whole a greater implementation (of programs) in decentralized countries (60%), in countries with a NHS model (68%), and also in countries with a strong/medium PC (67%: 42% strong and 25% medium). In this respect, there are no significant differences between all programs for adult people aged 18+ and those with explicit focus on older people aged 65+ (Table 1). For this reason, the analyses which follow will target all 85 programs using technological care solutions. Anyway, we provide also some general insights on older people when relevant.

### 3.3. General Aspects of Programs Using at Least One eHeath Tool and Health System Characteristics

With regard to general aspects of programs (Table 2), the main significant objectives were increasing multidisciplinary collaboration (85% overall, 91% in countries with strong/medium PC, *p* = 0.039) and improving care coordination (72% overall, 79% in countries with NHS, *p* = 0.027; 82% in countries with strong/medium PC, *p* = 0.004). Main organizations and care providers significantly involved were, respectively, PC (71%) and GPs (80%) and both in programs which were identified in decentralized and SIS/TC countries, with strong/medium PC.

Other less involved providers, e.g., districts/community nurses and home helps, showed greater significant rates in countries with strong/medium PC, whereas hospital/specialized nurses and physiotherapist/exercise therapist showed greater significant rates in countries with NHS. Among mapped programs, 62% operated both at policy/managerial and patient level, and this regarded 75% of those found in countries with strong/medium PC (*p* = 0.001). Initiatives were on the whole implemented mainly regionally/locally (78%), and few of them showed a national/international dimension (22%). In this respect, significant differences emerged with regard to the strength of PC and level of (de)centralization. On the whole, the mapped programs adopting eHealth were mainly local and/or regional, and these with greater intake in decentralized countries and with strong/medium PC. Conversely, programs adopting eHealth were mainly implemented nationally/internationally in centralized countries and with weak PC (*p* = 0.001).

Finally, the main types of care/support for MM patients addressed by the programs were medical care (79%) and prevention (68%), without significant differences regarding health system characteristics. Less addressed types of care and support provided by the programs were significantly mainly provided in countries with NHS (74% for nursing care, *p* = 0.022), decentralized countries (73% for adherence to medication, *p* = 0.034), and countries with strong/medium PC (71% for coordination of medical services, *p* = 0.039).

### 3.4. Categories of eHealth Tools Adopted in the Programs and Health System Characteristics

#### 3.4.1. Healthcare Management (Communications between Providers)

With regard to categories of eHealth tools adopted in the programs (Table 3), all programs used at least one eHealth tool of this group. Within this group, we also found the three most used eHealth applications, e.g., EHRs (71%), registration databases with patients' health data that can support decision making (about 64%), and digital communication between care providers (47%), but with no significant differences concerning health system characteristics of countries. Anyway, data showed that in particular EHRs were mainly used in decentralized countries with NHS and strong/medium PC, whereas EHRs were mainly planned in centralized and SIS/TC countries, with weak PC. It has to be highlighted *(data not shown in Tables)* that the three most used eHealth applications mentioned above showed a slightly higher intake among programs focusing on the elderly (respectively, 76%, 67%, and 52%). Significant differences regarding the greater use of eHealth, according to health system characteristics of countries, were conversely found with regard to the eHealth tool less adopted in the group *Healthcare Management* as follows: eReferral systems and electronic reminders for providers (respectively, 41%, *p* = 0.048, and 35%, *p* = 0.036, in programs implemented in decentralized countries) and PHRs (25%, *p* = 0.050, in programs implemented in countries with strong/medium PC).

#### 3.4.2. Remote Consultation, Monitoring, and Care (Interaction between Patients and Health Professionals, including ePrescription)

On the whole, 68% of programs used at least one tool of this group, and we found a greater significant use of this entire group of applications in decentralized than in centralized countries (respectively, about 77% and 56%, *p* = 0.046). No further significant values emerged with regard to health system characteristics of countries.

#### 3.4.3. Health Data Analytics (Systems for Analysing Clinical Data of Patients)

Among programs, 40% adopted one application of this group, in particular computerized decision supports (35%). We found significant differences for the entire group and for single eHealth applications included, with regard to health system characteristics of countries, i.e., greater adoption in decentralized countries and in countries with strong/medium PC.

#### 3.4.4. Self-Management (of Patients to Live More Independently)

The eHealth solutions included in this group, i.e., supporting self-management of patients (e.g., electronic reminders and computerized tools), could be greatly beneficial to meet the very complex health needs of patients with MM. However, these tools are scarcely used by the programs and not yet widely adopted. The whole group of applications is indeed used by 39% of integrated care programs, and in particular online decision supports were the least frequently adopted tools (about 4%). Considering the characteristics of health system of countries, only a significant difference emerged with regard to the whole group of tools, with programs adopting such applications being mainly implemented in countries with NHS *vs.* SIS/TC (46% and 22%, *p* = 0.039).

### 3.5. Potential Benefits and Barriers of/for Adoption of eHealth in the Programs and Health System Characteristics

Potential benefits of using eHealth in the programs, as reported/perceived by program managers, are reported in Table 4. On the whole, 95% reported improvements in management of care, 93% in care integration, and 86% in quality of care provided. No significant differences emerged with regard to health system characteristics of countries. Benefits were also reported, with slightly higher percentages, for programs targeting the elderly (*data not shown in Tables*). Concerning barriers hampering the use of eHealth tools in integrated care programs, various significant differences among groups of countries emerged, and in great part, they put in evidence a worse generalized perception (greater rate of agreeing by program mangers) in centralized countries and with weak PC, regarding mainly inadequate funding (respectively, 87%, *p* = 0.001, 83%, *p* = 0.011) and inadequate technical ICT support (respectively, 78%, *p* = 0.004, 78%, *p* = 0.012). Further (minor) significant barriers emerged again in centralized countries (inadequate ICT infrastructure and lacking technological skills among patients) and with weak PC (inadequate legislative framework). Regarding programs implemented in SIS/TC countries, the lack of skills and resistance among providers, resistance by patients, and obstacles linked to privacy issues emerged as significant barriers.

### 3.6. Insights from Case Studies of HPPs Adopting eHealth and Health System Characteristics

The general results reported above and regarding a greater implementation of integrated care programs adopting eHealth in decentralized countries, with NHS and strong/medium PC, and, conversely, a greater perception of barriers hampering this adoption in centralized and SIS/TC countries, with weak PC, are also confirmed by qualitative information/data gathered during the site visits of the HPPs we selected among the mapped programs. We found indeed that (Table 5), among the six (out of eight) HPPs using eHealth that we analysed for this paper, three initiatives were implemented in countries showing the three health system characteristics mentioned above, such as decentralized countries, with NHS, and with strong PC. These programs are the following: the Clinic for Multimorbidity and Polypharmacy in Denmark [72], the POTKU project (Putting the Patient in the Driver's Seat in Finland [73], and the Strategy for Chronic Care in the Valencia region in Spain [74]. Regarding the other HPPs using eHealth, we also noticed that when they are implemented in SIS countries, PC is anyway strong/medium, i.e., the INCA program in The Netherlands [75] and the Gesundes Kinzigtal program in Germany [76]. Moreover, when the HPP is implemented in a centralized country and with weak PC, the national health model is anyway a NHS, i.e., the TeleRehabilitation program in Cyprus [77].

Regarding qualitative information from site visits on barriers for using eHealth in the programs (*infos not shown* and drawn from the sources indicated in Table 5), we found some issues confirming quantitative analyses. Inadequate funding, that is the main obstacle in centralized countries and with weak PC, emerged indeed in the TeleRehabilitation program (in Cyprus). We also found resistance by patients to adopt eHealth, i.e., a barrier indicated in particular with regard to SIS countries, in the Gesundes Kinzigtal program (in Germany), and lacking technological skills among patients, another barrier indicated in particular with regard to centralized countries, in the INCA program (in The Netherlands). Qualitative information from site visits on benefits of using eHealth in the programs (*infos not shown* and again drawn from the sources indicated in Table 5) also confirmed results found in quantitative data (exposed above), such as a generally diffused perception of them among program managers, independently from characteristics of health systems of countries. We found indeed benefits reported in decentralized countries, with NHS, with strong PC, as good coordination/integration of care due to the sharing of EHRs among physicians and patients in the Clinic for Multimorbidity and Polypharmacy, due to advanced decision support systems (DSSs) in the Strategy for Chronic Care and due to a computerized decision support e-tool for GPs in the POTKU project. We also found some perceived benefits regarding the other HPPs reported in Table 5, e.g., in countries where PC is weak (e.g., Cyprus), in SIS countries (e.g., Germany, Netherlands), and in centralized countries (e.g., Cyprus and the Netherlands). These benefits are again improvements of management processes in the Gesundes Kinzigtal program, where the physicians share EHRs; in the INCA program, with care profiles for patients that are accessible by professionals and patient; and with remote monitoring and therapies at a distance, thus reducing readmissions and being cost-effective, in th5e TeleRehabilitation program.

Further qualitative information (e.g., aim, main activities, and eHealth aspects) obtained from the site visits of HPPs in Denmark, Finland, and Spain, is reported in a separate publication [65] and also briefly in text boxes which are included in Text S1 in the Supplementary Materials.

## 4. Discussion

The ICARE4EU study explored, among others aspects, the diffusion in Europe of integrated care programs for MM adopting eHealth applications. The overall findings presented in this paper suggest the presence of a link between health system characteristics of participating countries and general aspects of these programs, categories of eHealth adopted, and related benefits/barriers.

### 4.1. Overall Picture Emerged from the Study

As a first result of our study, among 85 programs which use of at least one eHealth tool in 24 countries, 15 were identified in Spain; 7 both in Greece, Iceland, and Germany; 6 in Italy; and 5 in Finland. This distribution is somewhat/partially different from the one reported by previous literature, i.e., greatest eHealth adoption in Nordic European countries [32, 44]. The reason for this difference could be twofold. On the one hand, it might partly depend on personal knowledge of country experts and program managers selected for the ICARE4EU study, who in some cases might not have been aware of all integrated care initiatives operating in their countries (as explained better in Limitations of the paper). On the other hand, the abovementioned discrepancy might depend on the fact that, in great part of European countries, eHealth tools have been generally adopted by health systems but rarely included in integrated care programs/practices for people with MM, as those mapped during our project [65]. Conversely, both our study and previous research findings [43, 78] put in evidence the relatively large number of health programs using ICT tools and identified in Spain that is a Southern European country. This context is probably due to the circumstance that Spain has 17 regions/autonomous communities and governance of health care delivery is provided at a regional level [79], thus leading to a considerable number of health programs on the whole. Moreover, Spain in recent years has greatly participated in European programs funding activities on ICT and ageing [37], throughout the “National Plans Research, Development and Innovation”, running from 2007 to 2012 [78]. In particular, in Spain, a great part of funding for remote monitoring projects is provided by the European Commission [80].

Moving specifically to health system characteristics of countries, we found on the whole a greater implementation of care programs adopting eHealth tools in decentralized countries, with a NHS model and with a strong/medium PC. These quantitative results were also confirmed/reinforced by the evaluation of the HPPs we selected among the mapped programs, which showed that three out of six good practices using eHealth were implemented in countries with such peculiarities.

The findings from our study are supported by previous authors. With regard to national health models, Codagnone and Lupiañez-Villanueva [32] found a positive link between eHealth adoption and NHS countries. Other authors [47] highlighted a greater implementation of eHealth, for instance ePrescribing, where the NHS model is provided, with a tax-based financing system, and with less barriers between different sectors of the health system, thus allowing a greater integration and coordination among professionals and services, also with the support of eHealth applications. Moreover, most NHS countries have carried out national ePrescribing projects for many years, whereas many SIS countries and TCs were at the beginning in this respect. The same authors [47] also showed that funding and incentives of healthcare operating in these nations seem more favorable to eHealth adoption than those available in SIS countries. In NHS countries, it is also effective the becoming “mandatory” obligation for GPs to adopt eHealth for administrative tasks, and the fact that a single authority or few institutions manage (in most cases) the national health information systems represents a key factor.

In accordance with our results, previous literature also highlighted that the adoption of eHealth seems more facilitated in decentralized health systems, where local governance supports intersectoral integration [52]. On the whole, financial, organizational, and political decentralization implies local autonomy and decisions according to the local preferences, with expected improvements in welfare efficiency and equity, and increased responsiveness of local authorities [81, 82]. In particular, some studies put in evidence how the organisational level can influence development and adoption of healthcare innovation/technologies; that is, organisations are supposed to assimilate innovations better if they are (among other factors) managed through decentralised decision making [83, 84]. Thus, decentralization seems to improve public service delivery also by allowing innovation [85]. It has anyway to be highlighted that, according to further studies, in countries with decentralized health systems, an official eHealth strategy with agreed common aims among different institutions is needed [57]. In other words, although in many cases digital health innovations are adopted at lower and decentralized levels in the health care system, implementation of digital health services on lower levels needs to be aligned with overall/central system goals [86].

The strength of PC is a further crucial aspect of health systems that emerged in our study as influencing the implementation of programs with eHealth solutions. Also, other studies report similar findings by evidencing how a strong PC system seems linked to innovative care opportunities offered by technology [49] and how eHealth tools in turn can improve PC consultations [87]. Recently, the European Commission [88] presented country-specific recommendations regarding more sustainable and innovative health systems by means of strong/well-performing PC. The strength of PC is indeed different across countries due to variation in political will, social-cultural values, policies, and healthcare system type; thus, country-specific strategies to develop PC are need [49, 89]. Consequently, specific country factors, such as organization and legislation of national eHealth services, play a role for eHealth adoption itself [47]. Further literature indicated that a strong PC, i.e., accessible, comprehensive, continuous, and coordinated [49, 90], can support and strengthen, as backbone, well-performing health systems, where integration of care can be provided especially to patients with MM [91–93]. And in order to deliver integrated services, with provision of both health promotion and prevention within PC, health systems should adopt innovative eHealth solutions [30]. In this respect, also, relevantly, some results [68, 94] show how healthcare expenditure (that could sustain technological innovation) in European countries is significantly linked to the strength of PC process. It is also to highlight that when countries decentralize important PC functions, this might lead to a not “clear governmental vision” regarding the future direction of PC ([95], p. 171).

### 4.2. Main General Aspects of Programs Adopting eHealth

We found that specific and main objectives of MM care programs using eHealth tools, such as increasing multidisciplinary collaboration and improving care coordination, were particularly operating on the whole in countries with NHS and countries with strong/medium PC. Batenburg ([96], p. 1541) analyzed in particular the link between a country's health system and PC strength, and he found that a great part of countries with a NHS model also show a strong/medium PC, this allowing an access to health care providers “at the right time and right place”. Further authors highlighted that a strong PC implies a coordinated and collaborative care [68], i.e., a context beneficial to multimorbid patients, and how health outcomes in multimorbid patients are consequently better in countries with a strong PC structure [51, 52], also with the support of technology [30]. Thus, objectives of MM care, such as a greater multidisciplinary and coordinated healthcare, can find a favorable substrate especially in countries with a NHS model and strong PC.

Main organization and care provider significantly more involved in our study were, respectively, PC services and GP, and in programs with eHealth which were identified in decentralized countries and in those with strong/medium PC. This context is not surprising, given that, in decentralized countries and with strong/medium PC, GPs are reported as main healthcare coordinators [96], the “core” providers of PC services, the key medical professional caring for (older) person with MM [97], and the most helpful service according to family carers opinion and also perceived as a real “support service” [98]. Moreover, in countries with a greater proportion of GPs, who can convince patients to use technology, eHealth could be more diffused. According to some authors, a positive cultural attitude towards new health technologies among physicians could play indeed a crucial role in making, e.g., telemonitoring more acceptable for their patients, especially for the elderly [65, 99]. PC services and GPs in our study are also significantly more involved in programs with eHealth which were identified in SIS/TC countries. This result does not seem in contrast neither with the fact that our results evidenced a greater involvement of PC services and GPs in countries with strong/medium PC nor with the fact that previous studies [96] found a link between strong PC and the NHS model in a country. The strength of PC, as depending on various factors (e.g., governance, economic conditions, accessibility, comprehensiveness, continuity, and coordination), including workforce development, is something different from a greater involvement of PC in eHealth programs. This seems rather to indicate that in SIS/TC countries, PC and GP represent basic pillars, given that other services/professionals are less involved, differently from NHS countries, where conversely the related percentages are higher. In particular, in our study, hospital/specialized nurses and physiotherapists/exercise therapists showed greater significant rates in countries with NHS. In this respect, other studies highlight that a strong PC structure is supported by national health workforce (HWF) developments [48] and that countries with a NHS model and strong PC (e.g., Denmark, Finland, the UK, and Spain) have highest levels of HWF planning [96]. A successful eHealth adoption requires in turn investment in both health and social care workforce [4].

By our findings, initiatives adopting eHealth were also on the whole significantly implemented mainly nationally/internationally in centralized countries and with weak PC and conversely mainly regionally/locally in decentralized countries and with strong/medium PC. This context could reflect the fact that in these latter countries, there are preconditions for supporting/allowing integrated care initiatives which are more adequate to local/specific needs, and which are more capillary and punctual. On the opposite, more general/national/international care programs, which are often related to temporary research projects and not based on local political/social policies, are more widespread in centralized countries and with weak PC, where the power for public health planning is not delegated to local/regional authorities but managed by the national/central government. Drawing on literature, we know that often adoption of eHealth applications ends when also related research projects are concluded, although successful [100], and that local health problems often require local solutions, with a crucial role of local research in providing adequate funds supporting local priorities [101]. In such a context, a strong PC represents the first “local” entry point into the “national” health system for the large majority of health needs [102], and the “promise” of decentralization seems potential for enabling local governments to provide care programs/health services more tailored on local interests/preferences, with a greater involvement of local communities [103]. Territorial approaches/perspectives also permit to reconnect decentralisation and development/innovation, with the support of “national decentralisation policies” ([104], p. 13).

The key roles of NHS model, decentralization of health system, and strength of PC are further confirmed by our results regarding some types of care and support which are provided by the programs adopting eHealth, e.g., nursing care, adherence to medication, and coordination of medical services. These were indeed significantly and, respectively, mainly provided in countries with NHS and in decentralized countries and with strong/medium PC. In this respect, we found partly support in previous literature with regard to the fact that a strong/integrated PC, in particular when continuous and coordinated [49], can provide some more specific types of care. Some studies of “exemplary”, high-performing, innovative PC practices [105–107] have put indeed in evidence a key role of nurses within the care team, including in particular management of chronic disease. Conversely, lack of coordination of care (e.g., in a weak PC system) can negatively impact for instance on medication adherence especially in case of patients with coexisting conditions and several medications prescribed by several physicians [108].

### 4.3. Categories of eHealth Applications

Our findings showed that all programs used at least one eHealth application of the group *Healthcare Management*, and mainly EHRs, registration databases with patients' health data, and digital communication between care providers. We found in particular that EHRs were the most used tools especially in decentralized countries with NHS and strong/medium PC (and mainly planned in centralized and SIS/TC countries, with weak PC), but with no significant difference with regard to health system characteristics of countries. These results on one side seem to confirm a wider adoption (and intention of adoption) of EHRs in many European countries, independently from specific aspects of healthcare systems, as emerged also from previous literature [31, 32, 43]. On the other side, our results regarding EHRs, although not significantly, however highlight decentralized countries, with strong PC, and with NHS model as “more advanced” and favorable context for supporting and implementing care programs with eHealth for patients with MM. In this regard, some authors [57] suggest that nationwide applications, e.g., national EHR systems, cannot be managed centrally, especially in large countries, and thus, the interoperability of regional systems is more successful in decentralized healthcare systems. Moreover, a strong PC seems to support a wider adoption of EHRs. High-performing health care systems, based on strong PC providing healthcare to multimorbid patients, require indeed monitoring them with the help of EHRs [109]. More recent findings [45] put in evidence that about 80% of PC practices were using an EHR across 15 EU countries, although there were wide variations. EHRs were especially used in all (or almost all) PC practices in Denmark, Estonia, Finland, Spain, and the United Kingdom, i.e., countries with strong PC (as they are in the classification by Kringos and colleagues [68].

Within the group *Healthcare Management*, and differently from the context of EHRs, our study found a significant and greater use of PHRs in programs which are implemented in countries with strong/medium PC. This characteristic of health systems seems thus crucial for promoting adoption of electronic/personal data of patients in integrated care programs for MM. According with Flaumenhaft and Ben-Assuli [110], governments try to balance the need to promote PHRs' use and the need to provide adequate protection of individual's medical information. In this respect, the recent European General Data Protection Regulation' (GDPR 2016/679, Reform 2018, European Union) [111], operating since May 2018, is requiring more strict protection measures regarding the handling of personal data, including sensitive health data among others [33]. For this aim, a strong PC system (e.g., with strong governance, accessibility, and coordination) seems however crucial.

With regard to other groups of tools, we found the following: a greater and significant use of the entire category *Remote Consultation*, *Monitoring*, *and Care* (tools for interaction between patients and health professionals) in decentralized countries; a significant use of the entire group and for single eHealth applications included *in Health Data Analytics* (systems for analysing clinical data of patients) in decentralized countries and with strong/medium PC; and a significant use of the whole group of tools for *Self-Management* (of patients to live more independently) in countries with NHS. To our knowledge, there is few literature supporting these specific findings, and conversely, we found much support from previous authors regarding on the whole how certain characteristics of health systems seem related to a more general adoption of eHealth applications, i.e., with decentralized health systems [57], with strong PC [49], and with a NHS model [32, 47]. However, some literature, regarding for instance online consultations, reported that in two decentralized countries such as Denmark and Finland, respectively, “the use of e-mail for consultations in general practice became mandatory in 2009”, and “e-mails between doctors and patients have been a routine part of care for over a decade” ([112], p 1). Other literature, regarding online decision supports used by health professionals, highlighted that these tools are specifically and increasingly important in PC for providing specific evidence on patients, e.g., for GPs [113], and thus, in this context, we could assume that a strong PC structure seems more favourable. Moreover, regarding tools for self-management, e.g., reminders by tablets, there are still few data on the structure/governance of the national healthcare systems and the related impact on frameworks of self-management support, and moreover, more research is needed for exploring “the optimal balance” for the delivery of self-management support through a national health system ([114], p. 8).

### 4.4. Benefits and Barriers of eHealth

Literature shows that both barriers/factors hindering and facilitators/factors enabling digital health implementation are crucial when planning healthcare [115, 116]. A large review on the implementation of eHealth in a wide range of healthcare systems ([117], p. 10) suggests that multiple factors were important and “no single factor was identified as a key barrier or facilitator” across different healthcare settings.

Our findings put in evidence some potential benefits of using eHealth in the integrated care programs for MCCs, as reported/perceived by program managers (e.g., improvements of care management/integration/quality). The fact that no eHealth benefit emerged as significant with regard to health system characteristics of countries clearly indicates that positive aspects of ICT use on healthcare are widely recognized in European countries. In this respect, almost all European member states of the WHO European Region participating in the eHealth survey 2015 [39] seem indeed to recognize benefits attributed to eHealth solutions which are implemented in the context of improved care management, thus facilitating the transition to patient-centered care models. Regarding interventions related to eHealth in PC and for MM, patient-centered care is indeed reported by Mangin and colleagues [118] as crucial for improving health outcomes and for integrated management of MCCs. Regarding specifically benefits of eHealth for MM, some authors state that the priority in high-performing healthcare systems, that is, to assure care coordination/integration [119], could be met better by eHealth application adoption and also that eHealth has potential for enhancing care integration/coordination among professionals/different providers, management processes, and continuity of care in all European countries [65].

Concerning significant barriers hampering the use of eHealth applications, we found greater rates of agreeing by program managers regarding mainly inadequate funding and inadequate technical ICT support in centralized countries and with weak PC. The lack of technological skills among patients and inadequate ICT infrastructure also emerged in centralized countries, whereas inadequate legislative framework similarly emerged in countries with weak PC. Privacy question and the lack of skills among providers were perceived as barriers in SIS/TC countries. These findings are on the whole consistent with previous literature [39, 117, 120]. In particular, several authors put in evidence how the adoption of EHRs in industrialized countries was hampered by security/privacy issues and complex legislation [121–123]. Regarding barriers in relation to some characteristics of health systems, the fact that we found them significantly more perceived in centralized and SIS/TC countries and countries with weak PC is in line with opposite findings from our study concerning a greater implementation of care programs adopting eHealth applications in decentralized countries, with a NHS model, and a strong/medium PC. Previous literature also indicate on the whole a wider and facilitated/not hampered adoption of eHealth innovations in European countries, e.g., due to available funding mechanisms, as linked to characteristics of healthcare systems [54, 55, 124, 125] In this respect, some authors showed that funding and incentive mechanisms of healthcare seem less effective in SIS countries than those operating in NHS countries [47]. Also, the degree of organization/centralization of health systems is reported as negatively impacting on interoperability of ICT applications in different countries [83, 84]. It is furthermore to highlight that government preferences for market legislation/forces may impact on the management of technology uptake in national healthcare systems, thus not leading to regulate eHealth infrastructure [126].

Also, qualitative information gathered during the site visits, to HPPs using eHealth, confirmed quantitative data on specific barriers hampering eHealth adoption, as perceived by program managers in centralized countries, in SIS/TC countries, and with weak PC. They further confirmed a generally diffused perception of benefits from eHealth adoption that is not linked to health system characteristics.

Our findings, also supported by previous literature, seem thus to confirm our hypothesis, such as that basic health system characteristics are relevant for the implementation of care programs for MM adopting eHealth in Europe.

### 4.5. Limitations

This study presents some limitations, as well as the overall ICARE4EU project [52, 65, 66]. Our overview of relevant programs in European countries is based only on the impact of eHealth technologies as perceived by service/program managers/leaders, without including reports from patients, caregivers, and care providers. We were also dependent on the personal expertise/knowledge (in some cases probably incomplete regarding all care approaches operating in their countries) of country experts and program managers participating in the survey. This may have influenced the number of mapped programs and of related countries where programs with eHealth were implemented (*n* = 24). This circumstance, in turn, may have impacted our analysis by health system characteristics of (groups of) countries that was based on classifications we built from literature (including 31 countries). No eligible program, according to our inclusion criteria, was indeed identified in Czech Republic, Estonia, Hungary, Poland, Romania, and Slovakia. Information on French programs was incomplete and thus excluded from the analysis. For United Kingdom, our study covered only England. Another weakness of the ICARE4EU study is the need to develop original survey questions, with the contribution of each partner regarding a specific expertise/research theme (patient centeredness, management practices and professional competencies, financing mechanisms, and use of eHealth technologies), due to not available validated questionnaires in all European countries (in 2014, year of data collection) to assess the practice characteristics of our interest [52]. Also, only eight selected programs could be visited in the scope of this project, and only six contained aspects of eHealth. Thus, we based our analyses mainly on data from the web survey and less on daily practice regarding eHealth adoption. The results of the qualitative data analysis provided however valuable insights in the current implementation of these good practices. Furthermore, we mapped eHealth aspects that were considered relevant for MM care, but comprehensiveness cannot be guaranteed. For the specific issue of eHealth, data collection instruments included indeed the most frequently used types of applications, without focusing on all available potential solutions, which would have required a more comprehensive data collection beyond the scope of the project [29]. Moreover, the design of the study and the lack of detailed information about the eHealth tools hampered a more in-depth analysis, such as inclusion of indicators at a macro level (e.g., the size or the economic situation of the country) and the extent or level of eHealth adoption (e.g., experimental/fully adopted, small/large scale). We recognize the limited validity and reliability of the dependent variable (i.e., number of programs adopting eHealth tools identified in a country), which could have produced an overestimation of the real eHealth adoption, where “small” or experimental tools are counted equally as “larger” and long-running tools and the single use for instance of EHR in a country is put on the same level of another more complex and innovative technological application. This may have further impacted on the relation between integrated care programs for MM using eHealth tools and characteristics of health systems, thus potentially affecting the cross-country comparison and limiting the conclusions drawn from the study, also with regard to policy implications. Such a context should lead to some caution in the interpretation of results. It is however to highlight that the ICARE4EU project did not aim to carry out a systematic mapping of good practices of integrated care initiatives for people with MM across Europe, and moreover, it was an exploratory study on the adoption of eHealth tools in the area of MM in Europe, and thus, we were interested in the use of at least one single tool. Despite all limitations listed above, influencing the findings and hampering their generalizability in particular with regard to the potential benefits of eHealth and despite the additional fact that the scale of the initiatives remained mostly local and/or regional, a large number of eligible eHealth programs were identified. It was thus an attempt to provide some insight into related current practice. Moreover, given that there are currently very few studies and data available on the specific issue of eHealth for MM care and with limited sample sizes, the 85 eHealth programs analyzed in this paper could contribute to some extent to have a European picture of the issue. Their knowledge could in turn support further development and implementation of MM care programs adopting eHealth in European countries.

## 5. Conclusions

The ICARE4EU findings, although in the light of some methodological limitations which allow only general considerations on eHealth adoption in Europe, seem to suggest on the whole a relation between implementation of integrated care programs for MM using eHealth tools and characteristics of health systems, i.e., a greater diffusion of such programs in decentralized countries, with a NHS model, and a strong/medium PC. Also, WHO recently stated that digital technologies within a country can strengthen health system depending on health system characteristics themselves, such as governance, legislation, policy, workforce, standards, and interoperability [127].

In our study, all programs adopted at least one eHealth tool of the group *Healthcare Management* and mainly EHRs in decentralized countries with NHS and strong/medium PC, although no significant difference emerged in this respect. With regard to other groups of tools, *Remote Consultation*, *Monitoring*, *and Care* and *Health Data Analytics*, and tools for *Self-Management*, the characteristics of healthcare systems mentioned above seem on the whole significantly more favourable for the diffusion of integrated care approaches adopting new digital health technologies. Conversely, barriers to eHealth adoption emerged significantly more perceived in centralized countries and with weak PC (e.g., inadequate funding and inadequate technical ICT support) and in SIS/TC countries (e.g., privacy issues). The fact that no eHealth benefit emerged as significant with regard to health system characteristics of countries clearly indicates that positive aspects of ICT use on healthcare are widely recognized across European countries. These general quantitative results were also supported by some qualitative information gathered during site visits to HPPs using innovative/digital applications.

The potential useful adoption of eHealth seems thus more facilitated in decentralized health systems, where local governance can allow integrated care initiatives which are more tailored on local/specific needs, with the help of new technologies. In particular, when patients, professionals, entrepreneurs, and government collaborate together, starting in local communities with few initial eHealth tools, the potential of eHealth can be “enforced” [100]. Furthermore, a greater adoption of eHealth seems to emerge where a NHS model are provided, that is with a tax-based financing system and with healthcare incentives which seem more favorable to eHealth implementation. Finally, also PC seems to have a key role, given that it can strengthen healthcare systems and can provide a more flexible access to services and integration across settings through technology [91, 128]. A strong PC system can facilitate for instance a comprehensive care coordination [129, 130], and this context influences the adoption of eHealth applications. Moreover, in order to support PC, available and accessible health technologies thus “offers an opportunity that cannot be missed” ([131], p. 1373), by expanding the range of addressed health conditions and available related treatments [91].

eHealth shows thus a general good potential, and in this regard, decentralized countries with NHS and strong/medium PC seem to represent the more adequate contexts. However, current population ageing, increasing prevalence of chronic diseases and MM, consequent raising healthcare costs, and “budgetary constraints” will require crucial adaptations of European health systems, in order to provide a more innovative, integrated, and patient-centered care [132]. Health systems need in particular to become more strong/resilient and sustainable, and in this respect, the digital innovation might represent a key factor to promote healthy ageing [45]. In particular, a “responsible innovation in health” (RIH) could reduce the cost of innovation itself and contribute to the sustainability of health systems ([133], p. 64). However, although the digital transformation of health services is a process influencing the performance of health systems themselves, it seems crucial to collect further robust evidence to evaluate cost-effectiveness of new eHealth solutions and “to strengthen the political choice” to implement them ([16], p.1). Moreover, it seems important to carry out further studies on complex needs of frail older people with MM and “digitally supported flexible and adaptive teamwork”, given the paucity of data in this respect ([134], p.1). More in-depth analyses are needed, keeping into consideration the differences among various eHealth aspects and categories. The development of scales or indexes able to assess more precisely the level of eHealth adoption could further support comparative studies, in order to achieve a more precise knowledge on the topic across Europe and to actually test the country-comparative expectations.

## Figures and Tables

**Table 1:** Programs adopting at least one eHeath tool by health system characteristics of countries (% of programs)^a^.

Health system characteristics	All programs % (*n*)	With explicit focus on older people 65+ % (*n*)	*p* value
National health model^b^	*N* = 84	*N* = 42	0.483^e^
National Health Service (NHS)	67.9 (57)	71.4 (30)	
Social Insurance System/Transition Countries (SIS/TC)	32.1 (27)	28.6 (12)	
SIS	21.4 (18)	16.7 (7)	
TC	10.7 (9)	11.9 (5)	
Strength of primary care (PC)^c^	*N* = 83	*N* = 42	0.531^e^
Strong/medium	67.5 (56)	64.3 (27)	
Strong	42.2 (35)	40.5 (17)	
Medium	25.3 (21)	23.8 (10)	
Weak	32.5 (27)	35.7 (15)	
Level of (de)centralization of health system^d^	*N* = 85	*N* = 42	0.595
Decentralized	60.0 (51)	57.1 (24)	
Centralized	40.0 (34)	42.9 (18)	

^a^For details on countries of each group cfr.  Measures section and Appendix A Table S1 (as Supplementary material) in this paper. ^b^Determined in 2007/2013, based on Codagnone and Lupiañez-Villanueva, 2013 [32], and Brennan and colleagues, 2015 [47]. Data on Switzerland (i.e., regarding one program with eHealth) is not included, given that this country was not classified by Codagnone and Brennan. ^c^Determined in 2009/2010, based on Kringos and colleagues, 2013 [68], and Detollenaere and colleagues, 2017 [50]. Data on Croatia (i.e., regarding two programs with eHealth) is not included, given that this country was not classified by Kringos and Detollenaere. ^d^Derived from descriptive data in countries' latest (in 2013, year when the ICARE4EU project was initiated) health system review published in the WHO, Health System in Transition series (years from 2008 to 2013) [69]. ^e^*p* values regard the integrated classification SIS/TC and strong/medium PC.

**Table 2:** Programs adopting at least one eHeath tool by general aspects of programs and health system characteristics of countries (% of programs).

General aspects of programs	National health model		*p* value	Strength of PC		*p* value	Level of (de)centralization		*p* value	All programs *N* = 85 % (*n*)
	NHS *N* = 57% (*n*)	SIS/TC *N* = 27 % (*n*)		Strong/medium *N* = 56 % (*n*)	Weak *N* = 27 % (*n*)		Decentralized *N* = 51 *%* (*n*)	Centralized *N* = 34 *%* (*n*)		
Main objectives^a^										
Increasing multidisciplinary collaboration	86.0 (49)	81.5 (22)	0.596	91.1 (51)	74.1 (20)	**0.039**	88.2 (45)	79.4 (27)	0.268	84.7 (72)
Improving patient involvement	78.9 (45)	66.7 (18)	0.225	80.4 (45)	66.7 (18)	0.172	80.4 (41)	67.6 (23)	0.182	75.3 (64)
Improving care coordination	78.9 (45)	55.6 (15)	**0.027**	82.1 (46)	51.9 (14)	**0.004**	76.5 (39)	64.7 (22)	0.238	71.8 (61)
Reducing hospital admissions	68.4 (39)	74.1 (20)	0.597	73.2 (41)	63.0 (17)	0.340	72.5 (37)	67.6 (23)	0.627	70.6 (60)
Decreasing/delaying complications	63.2 (36)	74.1 (20)	0.322	58.9 (33)	77.8 (21)	0.092	58.8 (30)	76.5 (26)	0.093	65.9 (56)
Reducing public costs	63.2 (36)	66.7 (18)	0.754	62.5 (35)	63.0 (17)	0.967	62.7 (32)	64.7 (22)	0.854	63.5 (54)
Improving accessibility of services	66.7 (38)	51.9 (14)	0.192	67.9 (38)	51.9 (14)	0.158	68.6 (35)	52.9 (18)	0.144	62.4 (53)

Organizations involved^a^										
Primary care	63.2 (36)	85.2 (23)	**0.039**	85.7 (48)	40.7 (11)	**<0.001**	88.2 (45)	44.1 (15)	**<0.001**	70.6 (60)
General hospital	66.7 (38)	55.6 (15)	0.324	57.1 (32)	74.1 (20)	0.135	60.8 (31)	64.7 (22)	0.715	62.4 (53)
University hospital	43.9 (25)	37.0 (10)	0.554	46.4 (26)	33.3 (9)	0.258	47.1 (24)	35.3 (12)	0.282	42.4 (36)
Health centre	38.6 (22)	37.0 (10)	0.891	42.9 (24)	25.9 (7)	0.135	41.2 (21)	32.4 (11)	0.411	37.6 (32)
Community/home care organization	40.4 (23)	29.6 (8)	0.342	44.6 (25)	25.9 (7)	0.101	45.1 (23)	26.5 (9)	0.082	37.6 (32)
Government	35.1 (20)	37.0 (10)	0.862	42.9 (24)	22.2 (6)	0.067	43.1 (22)	26.5 (9)	0.118	36.5 (31)
Policlinic/outpatient/ambulatory care	26.3 (15)	55.6 (15)	**0.009**	41.1 (23)	25.9 (7)	0.178	39.2 (20)	32.4 (11)	0.520	36.5 (31)

Care providers involved^a^										
General practitioner (GP)	73.7 (42)	92.6 (25)	**0.044**	91.1 (51)	59.3 (16)	**0.001**	88.2 (45)	67.6 (23)	**0.020**	80.0 (68)
Medical specialists	71.9 (41)	63.0 (17)	0.406	64.3 (36)	81.5 (22)	0.110	62.7 (32)	79.4 (27)	0.102	69.4 (59)
Districts/community nurses	57.9 (33)	48.1 (13)	0.402	71.4 (40)	25.9 (7)	**<0.001**	66.7 (34)	38.2 (13)	**0.010**	55.3 (47)
Hospital/specialized nurses	66.7 (38)	29.6 (8)	**0.001**	50.0 (28)	66.7 (18)	0.152	45.1 (23)	70.6 (24)	**0.021**	55.3 (47)
Physioterapist/exercise therapist	56.1 (32)	29.6 (8)	**0.023**	44.6 (25)	55.6 (15)	0.351	45.1 (23)	50.0 (17)	0.657	47.1 (40)
Home helps	38.6 (22)	37.0 (10)	0.891	48.2 (27)	22.2 (6)	**0.023**	47.1 (24)	26.5 (9)	0.056	38.8 (33)
Social workers	42.1 (24)	29.6 (8)	0.271	39.3 (22)	37.0 (10)	0.844	35.3 (18)	41.2 (14)	0.583	37.6 (32)

Integration level			0.771			0.318			0.332	
Small-scale (pilot) program	22.8 (13)	29.6 (8)		21.4 (12)	33.3 (9)		21.6 (11)	32.4 (11)		25.9 (22)
Well-established and comprehensive program	29.8 (17)	29.6 (8)		28.6 (16)	33.3 (9)		27.5 (14)	32.4 (11)		29.4 (25)
Fully integrated in the regular healthcare system	47.4 (27)	40.7 (11)		50.0 (28)	33.3 (9)		51.0 (26)	35.3 (12)		44.7 (38)

Operational level			0.340			**0.001**			0.140	
Only at level of policy/management	5.3 (3)	0.0 (0)		1.8 (1)	7.4 (2)		2.0 (1)	5.9 (2)		3.5 (3)
Only at level of daily patient care	36.8 (21)	29.6 (8)		23.2 (13)	59.3 (16)		27.5 (14)	44.1 (15)		34.1 (29)
Both (policy/management—patient care level)	57.9 (33)	70.4 (19)		75.0 (42)	33.3 (9)		70.6 (36)	50.0 (17)		62.4 (53)

Adoption level			0.120			**0.001**			**0.001**	
Local	24.6 (14)	25.9 (7)		25.0 (14)	25.9 (7)		19.6 (10)	35.3 (12)		25.9 (22)
Regional	38.6 (22)	25.9 (7)		46.4 (26)	11.1 (3)		51.0 (26)	8.8 (3)		34.1 (29)
Local/regional, as part of a national program	10.5 (6)	33.3 (9)		19.6 (11)	14.8 (4)		21.6 (11)	11.8 (4)		17.6 (15)
National	15.8 (9)	7.4 (2)		3.6 (2)	29.6 (8)		3.9 (2)	26.5 (9)		12.9 (11)
National, as part of international programs	5.3 (3)	7.4 (2)		3.6 (2)	11.1 (3)		2.0 (1)	11.8 (4)		5.9 (5)
Inter-/supranational	5.3 (3)	0.0 (0)		1.8 (1)	7.4 (2)		2.0 (1)	5.9 (2)		3.5 (3)

Geographical coverage			0.822			0.429			0.339	
Only rural	5.3 (3)	3.7 (1)		3.6 (2)	7.4 (2)		2.0 (1)	8.8 (3)		4.7 (4)
Only urban	10.5 (6)	14.8 (4)		10.7 (6)	18.5 (5)		13.7(7)	11.8 (4)		12.9 (11)
Both rural and urban areas	84.2 (48)	81.5 (22)		85.7 (48)	74.1 (20)		84.3 (43)	79.4 (27)		82.4 (70)

Types of care and support provided by programs^a^										
Medical care	78.9 (45)	77.8 (21)	0.903	82.1 (46)	70.4 (19)	0.223	78.4 (40)	79.4 (27)	0.914	78.8 (67)
Prevention/delay of deterioration	66.7 (38)	74.1 (20)	0.493	62.5 (35)	81.5 (22)	0.081	64.7 (33)	73.5 (25)	0.392	68.2 (58)
Nursing care	73.7 (42)	48.1 (13)	**0.022**	71.4 (40)	59.3 (16)	0.268	66.7 (34)	64.7 (22)	0.852	65.9 (56)
Lifestyle and health behaviour	63.2 (36)	74.1 (20)	0.322	66.1 (37)	66.7 (18)	0.957	64.7 (33)	67.6 (23)	0.779	65.9 (56)
Adherence to medication	63.2 (36)	66.7 (18)	0.754	66.1 (37)	59.3 (16)	0.545	72.5 (37)	50.0 (17)	**0.034**	63.5 (54)
Medical treatment interventions	66.7 (38)	59.3 (16)	0.508	57.1 (32)	74.1 (20)	0.135	56.9 (29)	73.5 (25)	0.118	63.5 (54)
Coordination of medical services	66.7 (38)	51.9 (14)	0.192	71.4 (40)	48.1 (13)	**0.039**	66.7 (34)	55.9 (19)	0.315	62.4 (53)

^a^Only items with higher % (first seven main %); multiple answers were allowed. Each item had a yes/no format.

**Table 3:** eHealth tools (at least one) adopted in the programs, by categories (single tools and groups) and health system characteristics of countries (% of programs)^a^.

eHealth tools	National health model		*p* value	Strength of PC		*p* value	Level of (de)centralization		*p* value	All programs *N* = 85 % (*n*)
	NHS *N* = 57 % (*n*)	SIS/TC *N* = 27 % (*n*)		Strong/medium *N* = 56 % (*n*)	Weak *N* = 27 % (*n*)		Decentralized *N* = 51 % (*n*)	Centralized *N* = 34 % (*n*)		
*Remote Consultation, Monitoring, and Care* ^b^	*68.4 (39)*	*66.7 (18)*	*0.872*	*73.2 (41)*	*55.6 (15)*	*0.108*	*76.5 (39)*	*55.9 (19)*	***0.046***	*68.2 (58)*
Monitoring health status parameters by providers	31.6 (18)	37.0 (10)	0.620	33.9 (19)	29.6 (8)	0.695	37.3 (19)	26.5 (9)	0.300	32.9 (28)
Communication between care provider/patient^c^	33.3 (19)	18.5 (5)	0.160	28.6 (16)	29.6 (8)	0.921	27.5 (14)	32.4 (11)	0.627	29.4 (25)
Monitoring/interaction at distance (e.g., by video)	29.8 (17)	22.2 (6)	0.466	28.6 (16)	25.9 (7)	0.801	33.3 (17)	17.6 (6)	0.111	27.1 (23)
Online appointment scheduling	26.3 (15)	25.9 (7)	0.970	30.4 (17)	14.8 (4)	0.127	29.4 (15)	20.6 (7)	0.363	25.9 (22)
Registration health status parameters by patients	24.6 (14)	25.9 (7)	0.893	21.4 (12)	25.9 (7)	0.648	19.6 (10)	32.4 (11)	0.182	24.7 (21)

*Self-Management* ^*b*^	*45.6 (26)*	*22.2 (6)*	***0.039***	*39.3 (22)*	*33.3 (9)*	*0.599*	*37.3 (19)*	*41.2 (14)*	*0.716*	*38.8 (33)*
Electronic reminders	29.8 (17)	18.5 (5)	0.271	26.8 (15)	22.2 (6)	0.654	27.5 (14)	23.5 (8)	0.686	25.9 (22)
Computerized self-management tools	28.1 (16)	14.8 (4)	0.183	23.2 (13)	22.2 (6)	0.920	21.6 (11)	29.4 (10)	0.411	24.7 (21)
Online decision supports	3.5 (2)	3.7 (1)	0.964	1.8 (1)	7.4 (2)	0.199	3.9 (2)	2.9 (1)	0.810	3.5 (3)

*Healthcare Management* ^*b*^	*100.0 (57)*	*100.0 (27)*	—	*100.0 (56)*	*100.0 (27)*	—	*100.0 (51)*	*100.0 (34)*	—	*100.0 (85)*
Databases with patients' health data	70.2 (40)	51.9 (14)	0.102	60.7 (34)	66.7 (18)	0.599	68.6 (35)	55.9 (19)	0.232	63.5 (54)
ICT-based communication between care providers	50.9 (29)	37.0 (10)	0.235	51.8 (29)	40.7 (11)	0.345	47.1 (24)	47.1 (16)	1.000	47.1 (40)
Systems providing warning messages/information	40.4 (23)	25.9 (7)	0.198	41.1 (23)	22.2 (6)	0.092	43.1 (22)	23.5 (8)	0.064	35.3 (30)
eReferral systems	38.6 (22)	22.2 (6)	0.137	39.3 (22)	18.5 (5)	0.058	41.2 (21)	20.6 (7)	**0.048**	32.9 (28)
Electronic reminders	28.1 (16)	25.9 (7)	0.837	32.1 (18)	18.5 (5)	0.194	35.3 (18)	14.7 (5)	**0.036**	27.1 (23)
PHRs			0.443			**0.050**			0.055	
Used	19.3 (11)	11.1 (3)		25.0 (14)	3.7 (1)		25.5 (13)	5.9 (2)		17.6 (15)
Planned	5.3 (3)	11.1 (3)		5.4 (3)	11.1 (3)		7.8 (4)	5.9 (2)		7.1 (6)
EHRs			0.233			0.336			0.183	
Used	73.7 (42)	63.0 (17)		71.4 (40)	70.4 (19)		72.5 (37)	67.6 (23)		70.6 (60)
Planned	8.8 (5)	22.2 (6)		8.9(5)	18.5 (5)		7.8 (4)	20.6 (7)		12.9 (11)

*Health Data Analytics* ^*b*^	*42.1 (24)*	37.0 (10)	*0.659*	*51.8 (29)*	*11.1 (3)*	***<0.001***	*56.9 (29)*	*14.7 (5)*	***<0.001***	*40.0 (34)*
Computerized decision supports	35.1 (20)	37.0 (10)	0.862	44.6 (25)	11.1 (3)	**0.002**	49.0 (25)	14.7 (5)	**0.001**	35.3 (30)
Online decision supports	19.3 (11)	7.4 (2)	0.159	21.4 (12)	3.7 (1)	**0.037**	25.5 (13)	0.0 (0)	**0.001**	15.3 (13)

^a^Multiple answers were allowed. Each item had a yes/no format. ^b^At least one eHealth tool of the respective group (of tools) was adopted in the programs. ^c^Including ePrescription.

**Table 4:** Benefits and barriers of/for adopting eHealth tools (at least one) included in the programs by health system characteristics of countries (% agreeing in the programs)^a^.

Benefits/barriers of/for using eHealth tools	National health model		*p* value	Strength of PC		*p* value	Level of(de)centralization		*p* value	All programs % (*n*)
	NHS % (*n*)	SIS/TC % (*n*)		Strong/medium % (*n*)	Weak % (*n*)		Decentralized % (*n*)	Centralized % (*n*)		
Benefits	*N* = 42	*N* = 16		*N* = 38	*N* = 19		*N* = 37	*N* = 22		*N* = 59
Management of care	92.9 (39)	100.0 (16)	0.272	94.7 (36)	94.7 (18)	1.000	94.6 (35)	95.5 (21)	0.884	94.9 (56)
Integration of care	90.5 (38)	100.0 (16)	0.201	92.1 (35)	94.7 (18)	0.714	91.9 (34)	95.5 (21)	0.599	93.2 (55)
Quality of care	81.0 (34)	100.0 (16)	0.060	89.5 (34)	78.9 (15)	0.281	89.2 (33)	81.8 (18)	0.424	86.4 (51)
Cost efficiency	76.2 (32)	81.3 (13)	0.680	76.3 (29)	73.7 (14)	0.828	73.0 (27)	81.8 (18)	0.440	76.3 (45)
Quality of life	69.0 (29)	75.0 (12)	0.656	65.8 (25)	73.7 (14)	0.546	64.9 (24)	77.3 (17)	0.317	69.5 (41)

Barriers	*N* = 39	*N* = 18		*N* = 38	*N* = 18		*N* = 35	*N* = 23		*N* = 58
Inadequate funding	61.5 (24)	55.6 (10)	0.669	47.4(18)	83.3 (15)	**0.011**	42.9 (15)	87.0 (20)	**0.001**	60.3 (35)
Compatibility between different eHealth tools	53.8 (21)	55.6 (10)	0.904	50.0 (19)	61.1 (11)	0.436	48.6 (17)	65.2 (15)	0.212	55.2 (32)
Inadequate technical ICT support	53.8 (21)	61.1 (11)	0.607	42.1 (16)	77.8 (14)	**0.012**	40.0 (14)	78.3 (18)	**0.004**	55.2 (32)
Inadequate ICT infrastructures	56.4 (22)	50.0 (9)	0.652	44.7 (17)	66.7 (12)	0.125	42.9 (15)	69.6 (16)	**0.046**	53.4 (31)
Lack of skills among patients	46.2 (18)	61.1 (11)	0.294	44.7 (17)	61.1 (11)	0.252	40.0 (14)	69.6 (16)	**0.028**	51.7 (30)
Inadequate legislative framework	48.7 (19)	55.6 (10)	0.631	36.8 (14)	72.2 (13)	**0.013**	40.0 (14)	65.2 (15)	0.060	50.0 (29)
Lack of skills among providers	35.9 (14)	66.7 (12)	**0.030**	44.7 (17)	38.9 (7)	0.680	37.1 (13)	56.5 (13)	0.147	44.8 (26)
Uncertainty of cost efficiency	30.8 (12)	55.6 (10)	0.074	26.3 (10)	61.1 (11)	**0.012**	28.6 (10)	56.5 (13)	**0.033**	39.7 (23)
Privacy issues	23.1 (9)	61.1 (11)	**0.005**	21.1 (8)	55.6 (10)	**0.010**	22.9 (8)	52.2 (12)	**0.022**	34.5 (20)
Resistance by care providers	20.5 (8)	55.6 (10)	**0.008**	34.2 (13)	22.2 (4)	0.362	25.7(9)	43.5 (10)	0.159	32.8 (19)
Cultural resistance	17.9 (7)	38.9 (7)	0.088	28.9 (11)	11.1 (2)	0.140	25.7(9)	26.1 (6)	0.975	25.9 (15)
Resistance by patients	15.4 (6)	38.9 (7)	**0.049**	18.4 (7)	22.2 (4)	0.738	28.6 (10)	13.0 (3)	0.165	22.4 (13)

^a^Multiple answers/agreeing were allowed. Data were analysed as % of agreeing *vs* % of disagreeing for each benefit/barrier in the programs.

**Table 5:** HPPs adopting at least one eHealth tool by health system characteristics of countries.

Programs	Country	National health model^a^	Strength of PC^b^	Level of (de)centralization^c^
Clinic for Multimorbidity and Polypharmacy (Hujala and colleagues, 2015 [72])	Denmark	NHS	Strong	Decentralized
The POTKU project (Putting the Patient in the Driver's Seat (Hujala and colleagues, 2015 [73])	Finland	NHS	Strong	Decentralized
Strategy for Chronic Care in the Valencia region (Barbabella and colleagues, 2015 [74])	Spain	NHS	Strong	Decentralized
INCA model of integrated care for multimorbidity (Snoeijs and colleagues, 2015 [75])	The Netherlands	SIS	Strong	Centralized
The Gesundes Kinzigtal program (Struckmann and colleagues, 2015 [76])	Germany	SIS	Medium	Decentralized
TeleRehabilitation program (Barbabella and colleagues, 2015 [77])	Cyprus	NHS	Weak	Centralized

^a^Codagnone and Lupiañez-Villanueva, 2013 [32]; Brennan and colleagues, 2015 [47]. ^b^Kringos and colleagues, 2013 [68]; Detollenaere and colleagues, 2017 [50]. ^c^WHO, Health System in Transition series (years from 2008 to 2013) [69].

## Data Availability

Complete database is owned by partners. The dataset used for the aim of this paper is available from the first author (excluding the names of programs, and also excluding the names of countries as potentially identifying information, when in combination with basic characteristics of the programs), upon reasonable request to be determined by the ICARE4EU Consortium. All relevant data underlying the findings, which are described in the text (N values behind the measures reported), are however available within the paper.

## Supplementary Materials

Supplementary Materials Supplementary Table S1: health system characteristics and countries/groups of countries. Supplementary Text S1: text boxes with aim, main activities, and eHealth aspects of HPPs which were selected in Denmark, Finland, and Spain.
