# Supplementary material for: Integrated Care Programs for People with Multimorbidity in European Countries: eHealth Adoption in Health Systems
Source: Biomed Res Int. 2020 Apr 8;2020:9025326. doi: 10.1155/2020/9025326 (PMC7168691; doi:10.1155/2020/9025326)
Supplement: Supplementary Materials — Supplementary Table S1: health system characteristics and countries/groups of countries. Supplementary Text S1: text boxes with aim, main activities, and eHealth aspects of HPPs which were selected in Denmark, Finland, and Spain. [file 9025326.f2.zip › 9025326.f1.pdf]

Supplementary Table S1: Health system characteristics and countries/groups of countries

**Countries** (in bold ICARE4EU countries)	**National health model**[a]	**Overall Strength of PC**[b]	**Level of(de)centralization of health system**[c]
**Austria**	SIS	Weak	Decentralized
**Belgium**	SIS	Strong	Decentralized
**Bulgaria**	TC	Weak	Decentralized
**Croatia**	TC	(data not included)	Centralized
**Cyprus**	NHS	Weak	Centralized
Czech Rep.	TC	Medium	Decentralized
**Denmark**	NHS	Strong	Decentralized
Estonia	TC	Strong	Centralized
**Finland**	NHS	Strong	Decentralized
France	SIS	Medium	Decentralized
**Germany**	SIS	Medium	Decentralized
**Greece**	NHS	Weak	Centralized
Hungary	TC	Weak	Centralized
**Iceland**	NHS	Weak	Decentralized
**Ireland**	SIS	Weak	Centralized
**Italy**	NHS	Medium	Decentralized
**Latvia**	TC	Medium	Centralized
**Lithuania**	TC	Strong	Centralized
**Luxembourg**	SIS	Weak	Centralized
**Malta**	NHS	Weak	Centralized
**Netherlands**	SIS	Strong	Centralized
**Norway**	NHS	Medium	Decentralized
Poland	TC	Medium	Decentralized
**Portugal**	NHS	Strong	Decentralized
Romania	TC	Medium	Decentralized
Slovak Rep.	TC	Weak	Decentralized
**Slovenia**	TC	Strong	Centralized
**Spain**	NHS	Strong	Decentralized
**Sweden**	NHS	Medium	Decentralized
**Switzerland**	(data not included)	Medium	Centralized
Turkey	SIS	Weak	Centralized
**United Kingdom (only England in ICARE4EU)**	NHS	Strong	Centralized

[a] Codagnone et al., 2013 [32]; Brennan et al., 2015 [47]. NHS=National Health Service; SIS=Social Insurance System; TC=Transition Countries. Information on Switzerland was not collected because the related study was conducted in 31 countries (EU27 plus Croatia, Iceland, Norway, and Turkey). Data gathering process could start in November of 2012, and was mostly completed between January and March 2013. Croatia was included in the study although it was included in EU(28) in July 2013.

[b] Kringos et al. 2013 [68]; Detollenaere et al., 2017 [50]. Croatia was not collected, given that the survey kept in consideration EU27 within 2010 (plus Iceland, Norway, Switzerland and Turkey).

[c] WHO, Health System in Transition series (years from 2008 to 2013) [69]. http://www.euro.who.int/en/about-us/partners/observatory/health-systems-in-transition-hit-series (Countries' latest - in 2013 - health system review).
