# Supplementary material for: Integrated Care Programs for People with Multimorbidity in European Countries: eHealth Adoption in Health Systems
Source: Biomed Res Int. 2020 Apr 8;2020:9025326. doi: 10.1155/2020/9025326 (PMC7168691; doi:10.1155/2020/9025326)
Supplement: Supplementary Materials — Supplementary Table S1: health system characteristics and countries/groups of countries. Supplementary Text S1: text boxes with aim, main activities, and eHealth aspects of HPPs which were selected in Denmark, Finland, and Spain. [file 9025326.f2.zip › 9025326.f2.pdf]

Supplementary Text S1: Text boxes with aim, main activities and eHealth aspects of HPPs which were selected in Denmark, Finland and Spain.

Clinic for Multimorbidity and Polypharmacy, Denmark (Hujala et al., 2015 [72])

Aim: The Clinic for Multimorbidity and Polypharmacy in Denmark (established in 2012, Regional Hospital/Diagnostic Centre of Silkeborg – regional program) aims to support collaboration and integrate multidisciplinary primary healthcare teams (including medical doctor, nurse, pharmacist, physiotherapist, occupational therapist, relevant specialists, e.g. psychiatrist) managing patients with multimorbidity and receiving polypharmacy.
Activities: This results is deep assessment of the patient's disease status (including a review of their medication plan and follow-up recommendations), knowledge/information sharing among care professionals/specialist involved and GPs, and efficient use of resources.
eHealth: In particular the sharing of EHRs seems is allowed both to physicians and patients involved in the program, and a videoconference system has been tested (during the multidisciplinary team conference) to enable GP participation and joint decision-making. Moreover, a regional Electronic Patient Journal is available with information on patients (medication, blood tests, X-rays, blood pressure). GPs only have access to their own patients' information. Pharmacies have access to all electronic prescriptions (which have been used widely in Denmark since 2013).

The POTKU project (Putting the Patient in the Driver's Seat), Finland (Hujala et al., 2015 [73])

Aim: The POTKU project (regional program 2010–2014) in Finland aims to improve patient-centred care for people with a chronic disease (many with multimorbidity) seeking care from a local primary care centre. The project consisted of several sub-programs in five hospital districts in Middle Finland, covering 61 municipalities and about one million inhabitants.
Activities: This results in development of personalized health and care plan, of a pathway for people with multimorbidity, integration of care services, and supporting of self-management skills and patient education.
eHealth: This program promoted the collection/sharing of patient information by care professionals into EHRs, and a computerized decision support e-tool (Evidence-Based Medicine electronic Decision Support) for GPs (for care guidance, reminders and warnings) was developed. Furthermore, other eHealth tools were implemented (e.g. eOwnHealth, an electronic service that allows patients to access their laboratory tests and communicate with care professionals).

Strategy for Chronic Care in the Valencia region (Barbabella et al., 2015 [74])

Aim: The Strategy for Chronic Care in Valencia Region in Spain (regional program introduced in 2014) aims to develop an integrated care model/framework for multimorbid patients with chronic diseases, and in need of very complex care, needing thus chronic care or palliative care, who access hospital services and/or live in the community.
Activities: This results in collaboration/integration between hospital, primary, and community health services, stratification of patients according to morbidity profiles/risks, and monitoring of drug therapies and consumption by case managers, thus allowing the continuity of care with support to patients/carers/families by means of Information and communication technologies.
eHealth: ICTs represent an important pillar of the Strategy, by providing electronic tools for supporting care organisation and management, and in particular for stratifying population according to patient' morbidity profile and monitoring their drug therapies (e.g. to check adherence, to review medication and use of drugs). A computerized DSS for professionals connecting available clinical evidence on treatments/best practices with the complex profile of multimorbid patients was thus adopted. EHRs and electronic patient identifiers were used by all actors in the care network, as doctors, nurses, specialists, pharmacists.
